# Mesenchymal Stem Cells for Enhanced Healing of the Medial Collateral Ligament of the Knee Joint

**DOI:** 10.3390/medicina59040725

**Published:** 2023-04-07

**Authors:** Chul-Soo Lee, Ok-Hee Jeon, Seung-Beom Han, Ki-Mo Jang

**Affiliations:** 1 Department of Orthopaedic Surgery, Anam Hospital, Korea University College of Medicine, Seoul 02841, Republic of Korea; 2 Department of Biomedical Sciences, Korea University College of Medicine, Seoul 02841, Republic of Korea

**Keywords:** knee joint, ligament, ligament healing, medial collateral ligament, cell-based treatment, mesenchymal stem cell

## Abstract

*Background and Objectives*: The medial collateral ligament (MCL) is one of the major supporting ligaments of the knee joint, and MCL injuries are common where excessive valgus loading is applied to the knee joint. Although most MCL injuries can be treated conservatively, healing of the MCL can take several weeks to months. Furthermore, once injured, the biomechanical properties of the healed MCL differ from those of the native MCL, resulting in an increased risk of re-injury and chronic remnant symptoms. Mesenchymal stem cells (MSCs), owing to their therapeutic potential, have been investigated in various musculoskeletal injuries, and some preclinical studies regarding MSC-based approaches in MCL injuries have shown promising results. Despite satisfactory results in preclinical studies, there is still a lack of clinical studies in the orthopedic literature. This article describes the basic knowledge of the MCL, standard treatments for MCL injuries, and recent studies regarding the application of MSCs for enhanced healing of the MCL. MSC-based approaches are expected to be a potential therapeutic option for enhanced healing of the MCL in the future.

## 1. Introduction

The medial collateral ligament (MCL) is a major ligament of the knee joint. The MCL plays an important role in resisting valgus stress, rotational forces, and translational forces on the tibia as a primary static stabilizer. It is well known that the MCL is the most frequently injured ligament in the knee, accounting for up to 40% of all knee ligament injuries [1], and, based on a 10-year study of athletic knee joint injuries, 7.9% of all knee injuries [2].

To date, the optimal treatment for MCL injuries is based on injury severity and concomitant injuries to associated structures around the knee joint. However, in light of the established research results so far, it is well known that the recovered MCL inherently does not have the biochemical characteristics of the native MCL, regardless of non-operative or operative intervention. Accordingly, biological augmentation strategies have recently been explored to overcome these limitations.

The treatment of MCL injuries is evolving as more knowledge on the anatomy and biomechanics of the MCL, as well as the associated biochemical factors that promote the healing process, have been elucidated. Recently, investigations of biological agents for MCL injuries at the preclinical level have begun to receive attention and have shown promising outcomes, suggesting therapeutic potential for clinical applications in the near future [3,4,5]. Among the recent trials focusing on therapeutic approaches, studies using mesenchymal stem cells (MSCs) have consistently been conducted to enhance MCL healing. The purpose of this review is to provide a comprehensive understanding of MCL, including anatomy, biomechanics, and current treatment approaches and their limitations, and to introduce the latest research trends on MSC-based approaches as a potential agent to enhance biological healing of the MCL.

## 2. Anatomy of the MCL

**Figure 1:**
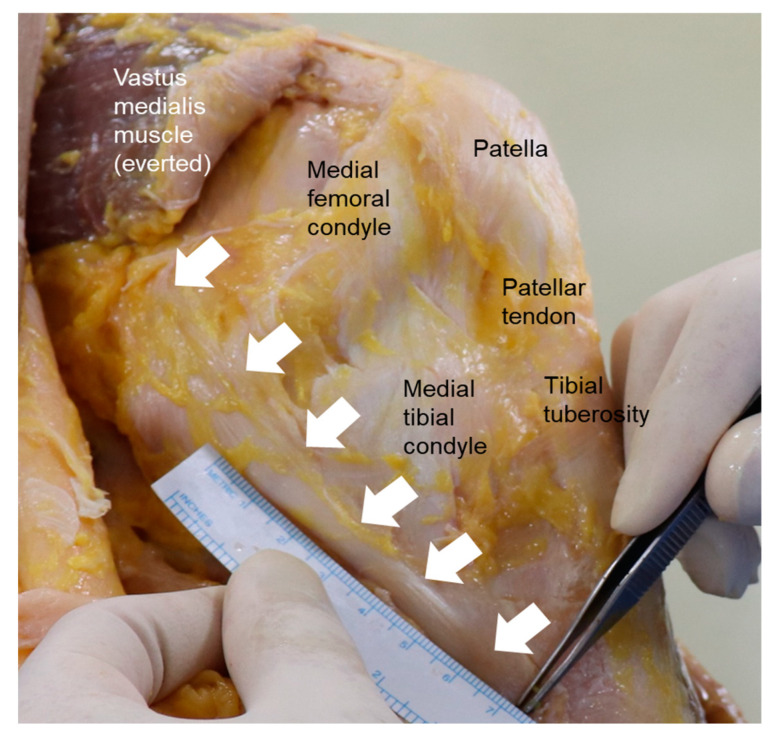
Anatomy of the medial collateral ligament (white arrows) and its surrounding structures.

The MCL is a capsuloligamentous complex located on the medial aspect of the knee joint (Figure 1). More than 40 years ago, Warren and Marshall demonstrated that the anatomical structures around the medial aspect of the knee joint are divided into three layers [6]. Layer I (the most superficial layer) comprises the fascia layer that surrounds the sartorius muscle, which partly forms the patellar retinaculum. Layer II (the middle layer) consists of the superficial MCL (sMCL), medial patellofemoral ligament, and posterior oblique ligament (POL). Layer III (the deepest layer) contains the deep MCL (dMCL), posterior medial capsule, and meniscotibial ligament.

The MCL is the largest ligamentous structure on the medial aspect of the knee joint, with a length of 90–110 mm, and consists of both superficial and deep components. The sMCL is a broad ligament composed of fibers that originate proximally from the medial femoral condyle, 1 cm anterior and distal to the adductor tubercle, and runs distally to the anteromedial aspect of the tibial crest, approximately 4.5 cm distal to the joint line posterior to the pes anserinus [6,7]. The dMCL, which is also called the midthird capsular ligament, is a thickening of the medial joint capsule that is made up of distinct meniscofemoral and meniscotibial portions. The posterior border of the dMCL blends along the central arm of the POL to form the posteromedial capsule. The meniscofemoral ligament is longer than the meniscotibial ligament, and originates from the femur, just distal to the sMCL origin, and inserts into the medial meniscus (MM). The meniscotibial ligament, which originates from the MM and attaches to the medial tibial plateau, is thicker and shorter [8].

## 3. Biomechanics of the MCL

The primary role of the MCL is to resist valgus stress and rotational forces [9,10]. It also acts as a secondary restraint to anterior tibial translation, particularly in anterior cruciate ligament (ACL)-deficient knees [11]. The MCL contributes to approximately 78% of the valgus restraining force at 25° of knee flexion, coordinating with the ACL, MM, posteromedial corner, and semimembranosus muscle. It also provides approximately 57% of the restraining force against valgus stress during knee extension [12]. Cutting studies have shown that the transected MCL causes 2° to 5° of medial laxity, or 3 to 5 mm of medial joint opening, when valgus stress is applied, whereas transection of both the MCL and posteromedial capsule causes 7° to 10° of medial laxity [7].

Several biomechanical studies have demonstrated that the amount of strain on the MCL differs according to the degree of knee flexion and the location (femoral origin, midsubstance, and tibial insertion) [13]. During knee flexion, different degrees of force are loaded on the anterior and posterior fibers of the MCL. The anterior fibers are stretched during knee flexion, whereas the posterior fibers remain relaxed. During knee extension, the anterior fibers are relaxed and the posterior fibers are stretched. The highest strain on the MCL is observed over the posterior aspect of the MCL near the femoral attachment at full extension. This finding correlates well with clinical data, suggesting that the femoral attachment site is the most commonly injured site in MCL injuries.

## 4. Etiology and Mechanism of MCL Injury

As the MCL is the most commonly injured ligament of the knee joint, accounting for up to 40% of all ligamentous knee injuries [1,14], injuries can occur during both contact and non-contact activities in any situation where excess valgus force is applied to the knee joint. Other injury mechanisms include external rotation pivoting injuries and direct blows to the anterolateral knee, as the lateral aspect of the knee is usually the most exposed region during sports activities.

The severity of MCL injury correlates with concomitant injuries to associated structures around the knee joint, including other ligaments, menisci, and other associated knee pathologies. Fetto and Marshall have reported associated injury rates as high as 78% in grade III MCL sprains [14]. The most common combined injury involves the MCL and ACL, comprising 7–8% of all ligamentous knee injuries [15].

## 5. Diagnosis of MCL Injury

The diagnosis of an MCL injury can be made by close history taking and rigorous physical examinations. A typical history is associated with recent trauma from a valgus blow to the lateral knee. Careful palpation along the entire course of the MCL is crucial. Physical examination for MCL injury is performed by applying a gentle valgus stress to the leg with 30° of knee flexion. Positive findings on physical examination include medial opening due to valgus stress and tenderness on the medial aspect of the knee joint. A comparison with the contralateral knee is necessary to determine the amount of joint line opening.

The extent of MCL injury is evaluated clinically by grade and degree; the former refers to the amount of joint line opening with valgus force and the latter refers to the quality of the end-point when laxity exists. The most commonly used classification system of MCL injuries is based on the American Medical Association classification [16], and the Hughston system was further developed [17]. Grade I is defined as a medial joint line opening of 0–5 mm, which corresponds to stretching and minor tearing of the MCL. This matches the definition of a first-degree sprain, in which there is tenderness over the MCL, but no instability. Grade II is defined as a medial joint line opening of 5–10 mm, which corresponds to a partial MCL tear. This matches the definition of a second-degree sprain, where there is increased valgus laxity with a firm end-point. Grade III is defined as a joint line opening of >10 mm, which corresponds to a complete MCL rupture. This matches the definition of a third-degree injury, where there is significant laxity with no accessible end-point [16,18].

In the clinical field, as well as within scientific papers, there is no standardized classification system describing the extent of MCL injury, and the terms “grade” and “degree” are often incorrectly or interchangeably used, causing problems with communication and the accuracy of analysis. Currently, most clinicians use a combination of elements consisting of clinical valgus laxity, endpoint quality, and magnetic resonance imaging findings [19].

## 6. Treatment of MCL Injury

### 6.1. Non-Operative Treatment

The therapeutic approach for MCL injuries generally depends on the extent of the injury and concomitant injuries. Non-operative treatment has been proposed as the mainstay of treatment for isolated grade I and grade II MCL injuries, focusing on early rehabilitation with range of motion (ROM) and progressive strengthening exercises. The main concept of rehabilitation is focused on early joint motion, as animal model studies have shown that immobilized joints can cause weaker ligament healing and poorer outcomes [20,21]. Additionally, functional bracing with a hinged knee brace can be used to prevent further valgus injury, while early ROM and weight bearing are encouraged as soon as pain has subsided.

Pharmacologic treatments, such as steroid injections and non-steroidal anti-inflammatory drugs (NSAIDs), are conventionally used because of their effectiveness in decreasing inflammation and pain from ligament injuries. However, given their inhibitory effects on ligament healing, resulting in the inferior histological, biochemical, and biomechanical properties of ligaments [22,23,24,25,26], many experts now caution against their use for treating ligament injuries, especially in athletes [27,28].

In a previous study, high school football players with grade I MCL injuries, treated non-operatively, returned to play by an average of 10.6 days after injury, and those with grade II MCL injuries were able to return by 19.5 days [29]. In another prospective observational study of 38 patients who were treated non-operatively for grade I or II MCL injuries, 74% returned to their preinjury activities and 95% returned to work 3 months later. At 4 years, Lysholm scores improved to 100, with only mild decreases in Lysholm scores and activity noted at the 10-year follow-up [30].

### 6.2. Operative Treatment

Surgical treatment of high-grade MCL injuries remains controversial. As the risk of accompanying injuries increases with the severity of MCL injuries, a thorough evaluation is necessary when selecting a treatment modality. With regard to the treatment of isolated grade III injuries, conservative treatment can be performed similarly to that for grade I and II injuries, and several studies have showed good clinical outcomes [31,32,33].

However, in severe injuries, damage to other structures must be considered. According to Fetto and Marshall, the risk of concomitant ligament injury was 78% for grade III MCL injuries [14]. The most common associated injury involves the MCL and ACL, accounting for 7–8% of all ligamentous knee injuries [15,34] and 70% of all multi-ligamentous knee injuries [35].

Surgical treatment is considered in some specific situations that involve complete ligament disruption. These include a large bony avulsion, tibial plateau fracture, complete tibial-side MCL avulsion, intra-articular entrapment of the end of the ligament, or anteromedial rotatory instability [36,37,38,39]. In addition, persistent valgus instability after conservative treatment in the injured knee could benefit from operative treatment.

## 7. Advent of MSCs as Cell Therapy for Enhanced MCL Healing

The majority of isolated MCL injuries are treated non-operatively due to their relatively good healing capacity; otherwise, they are often treated surgically for high-grade or combined injuries. However, once a ligament is injured, its biomechanical properties and function fail to return to those of the native ligaments, resulting in an increased risk of re-injury and chronic symptoms. Previous studies have reported abundant evidence that the inherent properties of the healing ligament remain compromised over the long term, characterized by less organization and composed of smaller collagen fibrils, and consequently exhibiting inferior mechanical strength [40,41,42,43]. As ligament laxity persists, the affected joint and other structures in and around the knee joint are exposed to the risk of further damage [44,45].

Considering the limited intrinsic healing potential, new approaches using biological augmentation have emerged as alternative means for ligament healing. These include gene therapy, biophysical stimulation, growth factors, and biologics to promote the quality of healing tissues and accelerate the healing rate without surgery, or as an adjunct to surgery [46,47,48]. The application of cells such as cultured fibroblasts, myoblasts, or MSCs as a biological vehicle [49,50,51] or growth factors have demonstrated some advanced results [52,53,54,55,56]. Among these, interest in cell therapy using MSCs continues to expand because of their ability to differentiate into several tissue cell types and modulate immune and inflammatory responses [57,58,59].

## 8. Recent Research on MSC Applications for MCL Injury

In recent years, MSCs have received increasing attention as a potential biological approach for MCL healing. Given the relatively high frequency of MCL injury and the subsequent growing interest in novel therapeutic approaches for its healing, the use of MSCs in the clinical field seems to be an attractive approach for regenerative treatment of ligament healing. From our research review, there were six studies which used MSCs for MCL healing (Table 1). These studies have only been conducted in preclinical animal model settings since 2002. The literature listed below is introduced chronologically, and reports several promising benefits of MSCs from different types of cell sources for enhanced MCL healing through their inherent potential, such as differentiation, vasculogenesis, and immune modulation.

In 2002, Watanabe et al. [60], in an in vivo animal study, investigated the morphology and distribution of donor bone marrow cells containing MSCs after simulated autologous transplantation. The MCL of the wild-type recipient rat was cut, where 1 × 10⁶ nucleated cells of bone marrow from the transgenic rat were injected. The results revealed that MSCs differentiated into fibroblast-like cells after transplantation into the healing ligament of the recipient. These cells were similar to those of the surrounding recipient MCL fibroblasts in morphology and continued to survive and migrate in the surrounding healing MCL until 4 weeks. The authors demonstrated that there is the potential for MSCs from the bone marrow to serve as therapeutic molecules, as well as to enhance MCL healing.

In 2008, Tei et al. [61] performed a preclinical study to clarify the role and efficacy of CD34+ cells in MCL healing. The authors demonstrated the therapeutic potential of circulating CD34+ cells for MCL healing by modulating the healing process through neovascularization. They examined the effects of human peripheral blood CD34+ cells on MCL injury in immunodeficient rats. The results revealed that the CD34+ group had a higher expression of human-specific markers for endothelial cells, such as Ulex europaeus lectin type 1 (UEA-1), increased neovascularization, and mRNA expression of vascular endothelial growth factor (VEGF), as well as higher gene expression of ligament-specific markers, than the other groups. The number of UEA-1+/HLA-ABC+ cells was significantly higher in the CD34+ group than in both the mononuclear cell (MNC) and control groups (CD34+, 119.5 ± 20.0; MNC, 54.0 ± 16.5; control, 0.0 ± 0.0 per mm^2^; *p* < 0.01, CD34+ vs. control; *p* < 0.05, CD34+ vs. MNC and MNC vs. control). Neovascularization (capillary profile number per mm^2^) was significantly enhanced in the CD34+ group compared to other groups (hCD34+, 301.0 ± 18.1; MNC, 209.5 ± 32.3; control, 108.6 ± 15.6; *p* < 0.01, CD34+ vs. control; *p* < 0.05, CD34+ vs. MNC and MNC vs. control). Furthermore, the histological and biomechanical properties of the early healing MCL were improved compared with those of the other groups. Histological evaluation with hematoxylin and eosin staining showed almost complete healing at week 2 and complete healing at week 4 in the CD34+ group. Biomechanical evaluation by failure load at week 2 was significantly higher in the CD34+ group than in the other groups (CD34+, 6734.766 ± 618.357; MNC, 3530.606 ± 1385.589; control, 3823.325 ± 95.538 mN; *p* < 0.05, CD34+ vs. MNC and hCD34 vs. control). These findings suggest that transplantation of circulating CD34+ cells promotes MCL healing by creating a favorable environment through neovascularization/angiogenesis.

In 2012, Nishimori et al. [62] investigated the therapeutic role of VEGF in the injured MCL of rats after muscle-derived stem cell (MDSC) transplantation. In the study, 5 × 10⁵ murine MDSCs transduced with the VEGF gene were transplanted into the MCL injury site of immunodeficient rats. Capillary density was significantly higher in the experimental group without significant differences in biomechanical properties compared to the other groups. In contrast, the group transplanted with MDSCs transduced with a VEGF-specific antagonist for the inhibition of angiogenesis (soluble fms-like tyrosine kinase-1) showed significantly decreased biomechanical properties in healing MCL. These results suggest that the transplantation of MDSCs into the injured MCL could accelerate the functional healing process, and MCL repair after MSC therapy might occur due to paracrine effects derived from the cells involved in angiogenesis.

In 2014, Saether et al. [63] examined the effect of MSC dosage on cellular response and cytokine profiles, as well as the different mechanical properties of MCL healing, using rat models. Two different cell doses (low dose: 1 × 10⁶ cells vs. high dose: 4 × 10⁶ cells) were administered to the MCL injury site. The high-dose group showed a significant decrease in M2 macrophages on days 5 and 14 postinjury and increased levels of several proinflammatory cytokines at day 5. The low-dose group revealed fewer M1 macrophages than the high-dose group on day 14, implying the immunomodulatory ability of MSCs on cellular response during MCL healing in a dose-dependent manner. In addition, mechanical tests demonstrated increased failure strength and stiffness in the low-dose group on day 14, suggesting that a lower dose of cells might be more effective in improving the functional properties of the injured MCL.

In 2015, Jiang et al. [64] evaluated the combined effect of MCL-tissue-derived MSCs (MCL-MSCs) and umbilical cord blood-derived CD34+ cells on MCL healing by gross observation, histological assessment, and biomechanical testing at 2 and 4 weeks using a rat model study. After MCL injury was treated by transplantation of MCL-MSCs and/or CD34+ cells, the results demonstrated that the group with concurrent administration of MCL-MSCs and CD34+ cells showed improved ligament healing, characterized by grossly diminished swelling, more regularly aligned fibers, increased type I collagen deposition and neovascularization, and higher tensile strength. The authors concluded that transplantation of a combination of MCL-MSCs and CD34+ cells could represent a promising therapeutic strategy for MCL injury.

In 2016, Saether et al. [65] tested whether primed MSCs enhanced MCL healing in a rat model. The authors elaborated on the experiment by priming MSCs for 48 h using polyinosinic acid and polycytidylic acid at a concentration of 1 μg/mL before application to the injured site of the MCL. Primed MSCs (1 × 10⁶ cells) in a carrier solution were transplanted into the MCL dissection site at the time of injury. Increased early endothelization, a higher number of M2 macrophages, interleukin-1 receptor antagonists, and procollagen 1a levels were observed compared with other groups, suggesting a more anti-inflammatory environment in the MCL healing process. Furthermore, both primed and unprimed MSCs co-localized with endothelial cells and pericytes, implying a supportive role in angiogenesis during the healing process.

As described above, there are several promising results in preclinical studies regarding the application of MSCs for the enhanced healing of MCL injuries. However, the current literature is limited to preclinical animal studies, with a lack of clinical evidence.

**Table 1:** Summary of recent studies regarding MSCs for enhanced healing of the MCL.

Ref.	Cell Source	Model	Results
Watanabe N. et al. [60]	BM-MSCs	Rat	At 3 days, transplanted MSCs from transgenic rats were evident in injured sites as well as the mid-substance of the recipient MCL. The MSCs continued to survive and were similar to recipient MCL fibroblasts morphologically at 28 days.
Tei K. et al. [61]	Peripheral blood CD34-positive cells	Rat	Local transplantation of human CD34+ cells in the ruptured MCL of immunodeficient rats revealed a higher expression of human-specific markers for endothelial cells, increased neovascularization and mRNA expression of VEGF, as well as higher gene expression of ligament-specific marker. In addition, histological and biomechanical properties were significantly enhanced in the CD34+ group compared to the other groups.
Nishimori M. et al. [62]	Muscle-derived stem cells	Rat	A total of 5 × 10⁵ MDSCs transduced with the VEGF gene was transplanted into the MCL injured site of immunodeficient rats. At 2 weeks, increased capillary density was observed in the MDSC-VEGF group compared with the other groups. In contrast, in a group transplanted with MDSCs transduced with a VEGF-specific antagonist (MDSC-sFLT1), decreased capillary density and significantly lower biomechanical properties were identified.
Saether E.E. et al. [63]	BM-MSCs	Rat	The higher-dose MSC group with 4 × 10⁶ cells showed decreased M2 macrophage level compared with the control groups on day 5 and 14 and increased level of several pro-inflammatory cytokines on day 5. On day 14, the lower-dose MSC group with 1 × 10⁶ cells showed lower M1 macrophage level than the higher-dose group. In a mechanical test on day 14, the lower-dose group showed an increased failure strength and stiffness compared to the higher-dose group.
Jiang D. et al. [64]	MCL-MSCs/CD34+cells	Rat	At 2 weeks, transplantation of combined MCL-MSCs and CD34+ cells in the MCL injured site showed a significant increase in capillary density than in the other groups. Failure load of the healing ligament was also superior in the combination treatment.
Saether E.E. et al. [65]	BM-MSCs	Rat	The primed MSCs for 48 h using polyinosinic acid and polycytidylic acid showed increased early endothelization, M2 macrophages, IL-1 receptor antagonists and procollagen 1a level compared with the other groups.

MSCs: mesenchymal stem cells; MCL: medial collateral ligament; BM-MSCs: bone-marrow-derived mesenchymal stem cells; mRNA: messenger ribonucleic acid; VEGF: vascular endothelial growth factor; MDSCs: muscle-derived stem cells; sFLT1: soluble fms-like tyrosine kinase-1; IL: interleukine.

## 9. Summary and Future Direction

The MCL is one of the major ligaments supporting the knee joint and is the most commonly injured ligament [1,66,67]. Considering the neglected injuries and the recent increasing popularity of sports participation among both the general public and professional athletes, the frequency of MCL injuries might be much higher than expected. Although the MCL has a relatively good healing capacity, several studies have demonstrated that healing ligaments are less organized with decreased mechanical strength, leading to an increased risk of reinjury and poor functional outcome [68]. For this reason, there is a consistent need for new strategies to expedite the restoration of the injured MCL to its normal condition. Among the various recently employed therapeutic approaches, the use of MSCs for enhancing MCL healing has been highlighted because of their extensive proliferative ability, multi-differentiative potential, and reparative capacities. Although insufficient, recent preclinical studies have shown promising results in enhancing MCL healing using MSCs. However, only limited preclinical studies have been conducted, and the precise mechanism by which MSCs enhance MCL healing is still unknown. Research regarding the proper cell source, cell dosage, and reproducible preparation process is also still in its infancy. Therefore, further extensive investigations on MSCs are necessary to elucidate the fundamental cellular and molecular mechanisms, as well as to validate their safety and efficacy in clinical settings for enhanced MCL healing.

## Data Availability

Not applicable.
